# Patients’ Perspectives About Factors Affecting Their Use of Electronic Personal Health Records in England: Qualitative Analysis

**DOI:** 10.2196/17500

**Published:** 2021-01-13

**Authors:** Alaa Abd-Alrazaq, Zeineb Safi, Bridgette M Bewick, Mowafa Househ, Peter H Gardner

**Affiliations:** 1 Division of Information and Computing Technology College of Science and Engineering Hamad Bin Khalifa University Doha Qatar; 2 Leeds Institute of Health Sciences School of Medicine University of Leeds Leeds United Kingdom; 3 School of Pharmacy and Medical Sciences University of Bradford Bradford United Kingdom; 4 Wolfson Centre for Applied Health Research Bradford United Kingdom

**Keywords:** electronic personal health records, tethered personal health records, patient portal, patient online, technology acceptance, technology adoption, qualitative research, mobile phone

## Abstract

**Background:**

General practices (GPs) in England have recently introduced a nationwide electronic personal health record (ePHR) system called Patient Online or GP online services, which allows patients to view parts of their medical records, book appointments, and request prescription refills. Although this system is free of charge, its adoption rates are low. To improve patients’ adoption and implementation success of the system, it is important to understand the factors affecting their use of the system.

**Objective:**

The aim of this study is to explore patients’ perspectives of factors affecting their use of ePHRs in England.

**Methods:**

A cross-sectional survey was carried out between August 21 and September 26, 2017. A questionnaire was used in this survey to collect mainly quantitative data through closed-ended questions in addition to qualitative data through an open-ended question. A convenience sample was recruited in 4 GPs in West Yorkshire, England. Given that the quantitative data were analyzed in a previous study, we analyzed the qualitative data using thematic analysis.

**Results:**

Of the 800 eligible patients invited to participate in the survey, 624 (78.0%) returned a fully completed questionnaire. Of those returned questionnaires, the open-ended question was answered by 136/624 (21.8%) participants. A total of 2 meta-themes emerged from participants’ responses. The first meta-theme comprises 5 themes about why patients do not use Patient Online: concerns about using Patient Online, lack of awareness of Patient Online, challenges regarding internet and computers, perceived characteristics of nonusers, and preference for personal contact. The second meta-theme contains 1 theme about why patients use Patient Online: encouraging features of Patient Online.

**Conclusions:**

The challenges and concerns that impede the use of Patient Online seem to be of greater importance than the facilitators that encourage its use. There are practical considerations that, if incorporated into the system, are likely to improve its adoption rate: Patient Online should be useful, easy to use, secure, and easy to access. Different channels should be used to increase the awareness of the system, and GPs should ease registration with the system and provide manuals, training sessions, and technical support. More research is needed to assess the effect of the new factors found in this study (eg, lack of trust, difficulty registering with Patient Online) and factors affecting the continuing use of the system.

## Introduction

### Background

Over the past 2 decades, there has been a rapid and widespread diffusion of electronic personal health records (ePHRs) in health care institutes [1]. The Markle Foundation defines ePHRs as web-based portals that enable users to access their medical records stored by their health care providers [2]. Other services can be added to ePHRs, such as booking appointments, requesting referrals, messaging health care providers, requesting medication refills, and educational materials [3,4]. Several benefits may be gained from using ePHRs, such as empowering patients [5,6], increasing their adherence to medication [7,8], improving their self-management [8,9], enhancing patient-provider relationships and communications [10,11], decreasing adverse events and allergic reactions [11,12], and avoiding duplicated tests [11,12].

General practices (GPs) in England started implementing ePHRs in 2003 when patients were enabled to access their full records through kiosks installed in some GPs. These kiosks allow patients to check their demographic information, consultations, test results, letters, and allergies [13].

In 2007, the National Health Service (NHS) offered patients in England access to their Summary Care Records (SCR) through HealthSpace [14-16]. HealthSpace is a secure web-based personal health record that has several functions: booking or canceling hospital appointments, recording and charting health indicators (eg, vital signs, weight, peak flow), calendar with email reminders, NHS address book, links to educational sources, secure messaging, and access to the SCR [15,17]. The SCR is a summary of key health information (allergies, adverse reactions, current medications, and main diagnoses) extracted from patient electronic medical records held by their general practitioners, and it is stored centrally and accessible by authorized NHS staff in urgent situations [14,16]. Because of the low adoption rate and technical issues, HealthSpace was shut down in December 2012 [18].

In 2015, the NHS implemented ePHRs under a program called Patient Online or GP online services, which enables users to book appointments, request prescription refills, and access coded information in their medical records such as demographics, medications, allergies, test results, problems list, immunizations, and medical and surgical procedures [19]. Currently, it is the largest ePHR in England, given that it has been implemented in more than 99% of GPs [19]. As the system is provided by different companies, it is called by different names such as Patient Access, Patient Services, The Waiting Room, and SystemOnline [19]. GP online services have been introduced in the United Kingdom at a time when funding for the NHS is under pressure. Given the context of austerity, individual practices have limited resources to support the rollout of GP online services.

### Research Problem and Aim

Despite the potential benefits of ePHRs, their adoption rate in England was only 28% by the end of June 2019 [20]. Identifying the factors affecting patients’ use of ePHRs is important to improve patients’ adoption and the implementation success of ePHRs [21-25]. A systematic review of 97 studies found that factors affecting patients’ use of ePHRs in England have not been examined, and there is a lack of qualitative studies (8%) in this topic [26]. Accordingly, this study aimed to explore patients’ perspectives of factors affecting their use of ePHRs (Patient Online) in England.

## Methods

### Data Collection

A cross-sectional survey was conducted between August 21 and September 26, 2017. In this study, a self-administered questionnaire was used to collect quantitative data through closed-ended questions and qualitative data through an open-ended question (Multimedia Appendix 1). The qualitative data provide the focus of this study. Note that the findings from the quantitative analysis of the survey data were presented in a previous paper [27]. The survey gained health research authority approval before starting data collection (The Research Ethics Committee reference number: 17/SC/0323).

### Sample

A convenience sample of patients was recruited from 4 GPs in West Yorkshire, England. Patients were eligible to participate if they (1) lived in England and were registered at 1 of the 4 GPs, (2) were aged 18 years or older, and (3) had not used Patient Online before (nonusers).

### Analysis

The qualitative data were analyzed using thematic analysis. Given the exploratory nature of this study, an inductive approach was used to generate themes directly from the data [28]. The analysis was performed following the steps proposed by Braun and Clarke [29]: (1) familiarizing with the data through scrutinizing and rescrutinizing the transcript; (2) coding data systematically; (3) generating subthemes and themes from codes; (4) checking the fit of those themes and subthemes to the original utterances and drawing an initial thematic map; (5) refining and regrouping some inappropriate codes and generating meta-themes from the themes for more granular grouping; and finally, (6) defining and naming subthemes, themes, and meta-themes. We followed the guidelines of Braun and Clarke, as these are considered the most systematic guide for conducting thematic analysis to date [30,31]. The analysis was carried out by the first author (AA), and the validity of codes and themes was checked by another author (BB). AA and BB discussed codes and themes. Where AA and BB had differing views on the code labels and/or thematic content, these discrepancies were resolved through discussion. In all cases, agreement was reached between AA and BB. Microsoft Excel was used to manage the analysis process.

## Results

### Collected Data

Out of the 800 eligible patients invited to participate in the survey, 624 (78%) participants completed the questionnaire. Of those participants, 136 (21.8%) answered the open-ended question. The 136 comments contained 221 utterances. A comment refers to the whole text written by a participant as a reply to our question, whereas an utterance refers to a part of the comment that has one idea or thought. In total, 3 of the 221 utterances were excluded because 2 utterances were illegible and the meaning of 1 utterance was not discernible. The final number of utterances included in the thematic analyses was 218. The excluded utterances were all part of longer comments, and for that reason, the final number of comments remained 136. Subsection 3.1 summarizes the characteristics of the respondents, and Subsection 3.2 presents the findings of the thematic analysis.

### Participants’ Characteristics

Table 1 summarizes the characteristics of the participants who answered the open-ended question and those who did not. Those who responded to the question had a mean age of 43.7 years (SD 18.3). More female participants answered the question than male participants (80/136, 58.8% were females). The majority of the respondents had a White ethnicity (107/136, 78.7%), had an income of less than US $40,000 per year (95/136, 69.8%), and had access to the internet (112/136, 82.4%). In terms of education, 39.7% (54/136) of the respondents had a bachelor’s degree or higher.

**Table 1:** Characteristics of respondents (n=136).

Characteristics		Value, n (%)
**Age (years), mean (SD)**		43.7 (18.3)
	18-24	19 (14.0)
	25-34	35 (25.7)
	35-44	23 (16.9)
	45-54	20 (14.7)
	55-64	17 (12.5)
	65-74	12 (8.8)
	≥75	10 (7.4)
**Sex**		
	Male	56 (41.2)
	Female	80 (58.8)
**Ethnicity**		
	White	107 (78.7)
	Asian	14 (10.3)
	Black	6 (4.4)
	Mixed	7 (5.1)
	Others	2 (1.5)
**Income (US $)**		
	<20,000	55 (40.4)
	20,000-29,999	24 (17.6)
	30,000-39,999	16 (11.8)
	40,000-49,999	9 (6.6)
	50,000-59,999	6 (4.4)
	60,000 or more	5 (3.8)
	Prefer not to say	21 (15.4)
**Education**		
	Up to secondary school	13 (9.6)
	Secondary school	31 (22.8)
	College/Diploma	38 (27.9)
	Bachelor’s degree	38 (27.9)
	Master’s degree	10 (7.4)
	Doctoral degree	6 (4.4)
**Internet access**		
	Yes	112 (82.4)
	No	24 (17.6)

### Findings of Thematic Analysis

**Figure 1:**
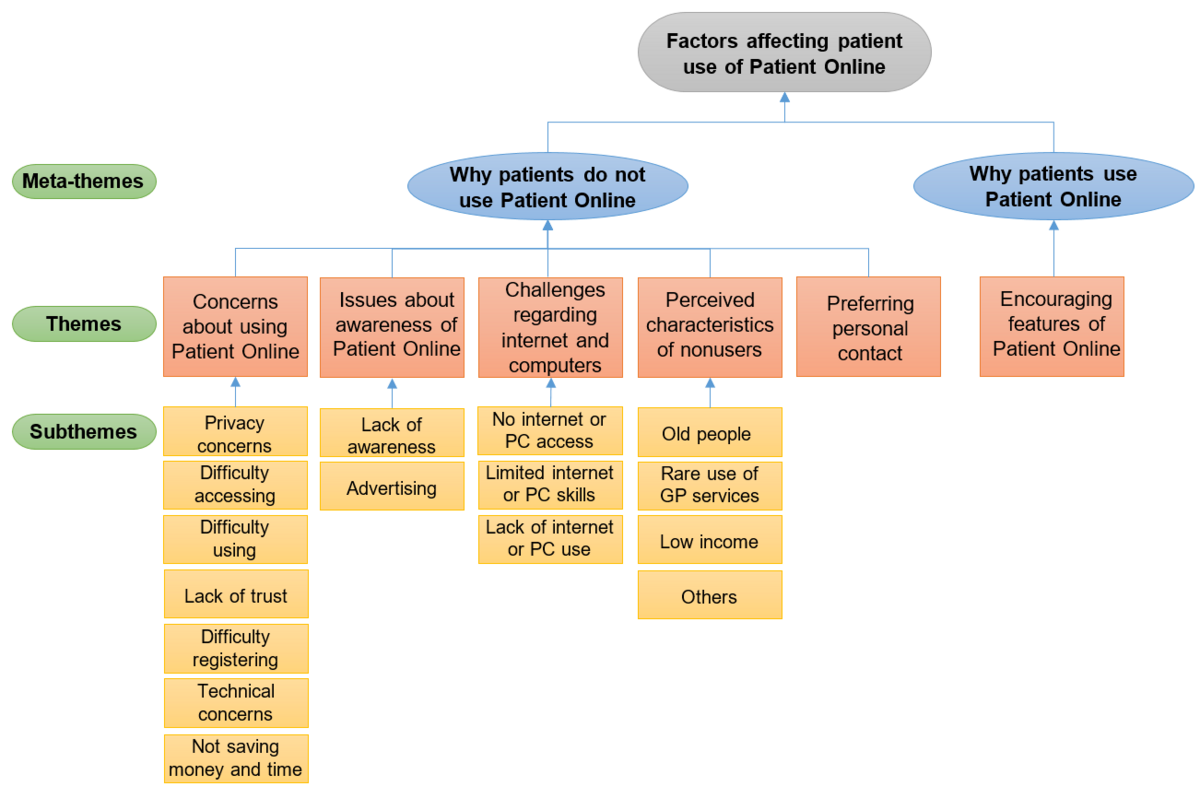
Thematic map. GP: general practice.

In total, 2 meta-themes were generated as a result of the thematic analysis. The first meta-theme consists of 5 themes and relates to utterances explaining why patients do not use Patient Online (Figure 1). The second meta-theme pertains to utterances about why patients use Patient Online, and it contains 1 theme: encouraging features of Patient Online. The following sections contain more details about all 6 themes.

#### Theme 1: Concerns About Using Patient Online

The first theme, concerns about using Patient Online, is made up of 7 subthemes: (1) concerns about privacy and security, (2) difficulty accessing Patient Online, (3) difficulty using Patient Online, (4) lack of trust in Patient Online, (5) difficulty registering, (6) technical concerns, and (7) the inability of Patient Online to save money and time (Figure 1).

The security and privacy of Patient Online was a major concern for respondents. Their concerns were attributed to the recent NHS hack attacks, worries that their data will be accessed by third parties, and uncertainty about the security measures of Patient Online:

I believe that Patient Online has/ will have too many privacy issues, look what happened when the NHS was hacked.

Participant #9

Only concern is confidentiality of System One as I am aware CIA [Central Intelligence Agency] are now using the system.

Participant #30

The second subtheme shows that difficulty accessing (logging in) the system can be a barrier to its use. The main reasons given for difficulty accessing Patient Online were the inability to find its URL link and forgetting passwords and log-in details:

I tried to use the system but I can never find the correct link...

Participant #120

...I always forget my password.

Participant #35

The third subtheme was generated from comments about difficulty using Patient Online. Although the previous subtheme reflects patients’ concerns about logging on to Patient Online, this subtheme represents their worries about using the system after logging in to it (ie, ordering prescriptions, managing appointments, checking their records). According to some respondents, these concerns are exacerbated when nobody can help in using the system:

I don’t know if this would be easy to use.

Participant #5

If people experience a difficulty and do not know where to find help, or who to ask, they may give up trying.

Participant #49

The fourth subtheme indicates that some patients did not trust Patient Online to do what they want it to do. They doubted that an appointment would actually be booked for them if they booked via Patient Online:

...I don’t trust the service.

Participant #9

...I am not sure I would entirely trust it...

Participant #123

Concerns with difficulty registering with Patient Online were brought together to form the fifth subtheme. These concerns were attributed to the fact that they have to visit the practice in person with their ID to be able to register to use the system. To ease the registration process, a respondent suggested that the registration to Patient Online becomes part of the patient registration in practice:

You also have to make a trip to the surgery with ID to be able to use the service.

Participant #28

I think more effort should be made to encourage patients to sign up for this, and the process should be more streamlined-perhaps done as a matter of course when registering.

Participant #7

The sixth subtheme encompasses utterances that show concern regarding the technical difficulties of Patient Online. Technical issues here refer to technical errors that people believe they will face when using Patient Online:

Technology goes wrong and does not tell you why.

Participant #58

The last subtheme brought together utterances from some respondents who were worried about the inability of Patient Online to save money and time. This is reflected in the utterances of the seventh subtheme, that is, respondents, especially those who live near the practice doubted that using Patient Online saves money and time:

In my experience many of these things do not end up saving people’s time and money. So I don’t think I’ll be using this except infrequently.

Participant #38

It would not save travel costs because I live next to it.

Participant #85

#### Theme 2: Issues About Awareness of Patient Online

The utterances in this theme suggest that if respondents had more knowledge or awareness about Patient Online, they would use it. This theme consists of 2 subthemes: lack of awareness of Patient Online and advertising about Patient Online. In the first subtheme, the respondents stated that the lack of knowledge about the system’s presence, what it is about, how to use, and how to access it was the main reason for not using it:

To be honest, I’ve never heard of Patient Online before and that may be why people haven’t used it.

Participant #88

Not been shown what it is about and how to use it.

Participant #80

In the second subtheme, several respondents attributed their lack of awareness of Patient Online to the lack of advertisement about it. For this reason, they acknowledged the essential role of the publicity of Patient Online in increasing people’s awareness of it:

It is not openly advertised in the surgery that Patient Online is available.

Participant #28

...may not be enough advertisement.

Participant #62

#### Theme 3: Challenges Regarding Internet and Computers

The third theme refers to issues regarding prerequisites for using Patient Online (ie, a computer and internet access). Respondents identified 3 challenges regarding the internet and computers, which form the 3 subthemes within this theme. The first challenge is the lack of internet or computer access. Many respondents attributed the nonuse of Patient Online to not having internet or computer access:

Those who don’t have access to the internet may not use it.

Participant #57

Although many respondents have access to the internet and computers, they have limited skills in using them, and this is the second challenge:

I can’t use a computer so I can’t use Patient Online.

Participant #2

The third challenge is the lack of use of internet or computers. This subtheme indicates that some users may have access to computers and internet and the required skills but do not frequently use them:

I do not use computers of any kind.

Participant #75

#### Theme 4: Perceived Characteristics of Nonusers

The fourth theme was generated from utterances about who is less likely to use Patient Online. The 3 main characteristics of nonusers were related to age, use of GP services, and income. These characteristics formed 3 subthemes, in addition to an extra subtheme that encompasses infrequently reported characteristics.

Age was the most commonly reported characteristic of nonusers. Respondents suspected the ability of older people to use Patient Online for different reasons: lack of computer and internet skills, lack of internet access, lack of awareness of how to use the system, lack of confidence in using it, lack of technology use, and their preference for face-to-face contact:

Elderly people may have no understanding or knowledge of how to use a computer or the internet.

Participant #69

Older people may not use it as they don’t have access to internet or know how to use services.

Participant #116

In the second subtheme, respondents attributed the nonuse of the system to lack of use of GP services in general, such as consultations and medications:

I’ve never used it as it’s rare that I attend the surgery and I’m not on any medication.

Participant #132

Low income formed the third subtheme. Respondents stated that people need enough income to have internet access or get training to be able to use computers and the internet:

I do not have enough income/benefits...

Participant #20

The last subtheme encompasses characteristics of other people who are more likely to be nonusers of Patient Online and those who live near the practice, illiterate people, people who cannot read in English, and people who forget to use Patient Online:

I would use Patient Online more often if I lived further away from the surgery.

Participant #15

I am not good at reading or spelling so online would not be good for me.

Participant #70

#### Theme 5: Preferring Personal Contact

Preferring personal contact was identified as the main justification for not using Patient Online. Patients prefer personal contact because they think it is more reliable, easier, provides an instant reply, and is important in urgent conditions:

It is more reliable to speak to someone directly about their medical records rather than using online.

Participant #29

Picking up the phone and speaking to someone is easier.

Participant #135

#### Theme 6: Encouraging Features of Patient Online

Within this theme, respondents identified features of Patient Online that may encourage them to use the system. One of the main features of Patient Online is that it is useful for different people, such as students, people with mobility needs, people who cannot reach the practice, and busy people:

I feel that it would be particularly useful for students.

Participant #63

Another feature mentioned by respondents is ease of access. Some respondents thought that Patient Online could be more accessible if it was a mobile app. It is noteworthy that mobile apps were not available for accessing GP online services at the time of data collection. Later, patients have been enabled to access GP online services via a mobile app called NHS App [32]:

A mobile application would be more accessible...

Participant #95

Respondents reported other features of Patient Online, which may encourage people to use it, namely, secure, quick, user-friendly interface, convenient, and less stressful:

If it is secure and fast then people will use it, I suppose.

Participant #68

If the interface is not user-friendly people might not be encouraged to use Patient Online.

Participant #82

## Discussion

### Principal Findings

The aim of this study is to explore why patients in England choose to use ePHRs. Participants identified one leading cause that encouraged them to use Patient Online, which relates to its features being useful, easy to access, secure, quick, user-friendly interface, convenient, and less stressful. However, patients identified many reasons for not using Patient Online, which were categorized into 5 themes: concerns about using Patient Online, lack of awareness of Patient Online, challenges regarding internet and computers, perceived characteristics of nonusers, and preference for personal contact.

In the first theme, concerns about using Patient Online, the most prominent reason for not using Patient Online was privacy and security concerns. This may be attributed to the fact that ePHRs typically contain personal and sensitive information, and patients have previously been shown to be concerned about the accessibility of these data [33]. The hack attacks that happened to the NHS 4 months before data collection may have exacerbated these concerns in this sample. This finding is consistent with the results of the quantitative data in the original study [27], where perceived privacy and security significantly affected patients’ intention to use Patient Online. This factor was also found in other quantitative studies [33-36] and qualitative studies [37-43].

Participants also raised their concerns about difficulty logging on to Patient Online because of losing its URL and forgetting passwords and log-in details. This issue posed a challenge for patients because they were given new complex passwords and usernames to access Patient Online. Although passwords can be changed through the system, usernames are fixed. This effect of difficulty accessing the system has also been demonstrated in several studies [41,44,45].

Other worries were reported about difficulty using Patient Online, especially when there is no one to help. This may be attributed to the fact that patients need adequate computer and internet skills to use Patient Online. They may also need to access it without any help from others to protect their privacy. This factor was also found in quantitative analysis in the original study [27], where effort expectancy (ie, ease of use) and behavioral intentions were significantly associated. Furthermore, numerous quantitative and qualitative studies have shown similar findings regarding this factor [37,38,40,41,45-47].

Participants expressed their concerns about the difficulty they experienced registering with Patient Online. Indeed, it could be argued that the process of registration with Patient Online is less flexible than several systems (eg, MyChart, PatientSite, My Health Manager, My Health at Vanderbilt), where patients can register with the system using email, websites, or phone and with no need to visit the practice. To the best of our knowledge, this factor was not found in previous studies. This may be because of the ease of registration with other systems.

The inability of Patient Online to save money and time was a concern for some participants, especially those who live near the practice. This concern may have made patients feel that Patient Online is not useful for them. Thus, this factor is related to perceived usefulness, which was the most influential factor according to the quantitative analysis in the original study [27]. The effect of this factor was also demonstrated by quantitative studies [46-49] and other qualitative studies [37,40,41,45,50].

Finally, 2 further concerns in this group were raised by participants, a lack of trust in Patient Online to book appointments or request medication refills and the technical issues that some patients reported when using Patient Online. To the best of our knowledge, neither of these factors have been reported in previous studies.

In the second theme, lack of awareness of different aspects of Patient Online was an influential factor in not using the system. Lack of advertising about Patient Online was the main reason for this lack of awareness. Although 3 of the 4 GPs had advertisements about Patient Online visibly displayed on screens or brochures in the waiting room during the study, some patients still reported a lack of awareness of the system. This factor was in line with the findings of previous quantitative studies [51,52] and qualitative studies [37,41,45,53].

With regard to the third theme, 3 challenges related to computers and the internet were identified. The first is the lack of internet or computer access. This factor was represented by the construct *facilitating condition* in the quantitative analysis, and it was found to significantly affect the actual use of Patient Online [27]. Furthermore, previous studies have shown a significant deleterious effect of a lack of internet [54-59] and computer access [38,46,52,57].

The limited skills in using the internet or computers was the second challenge in this group. This challenge may have produced reports that patients found Patient Online difficult to use. Hence, this factor is related to perceived ease of use (ie, effort expectancy), which was the most influential factor according to the quantitative analysis in the original study [19]. Numerous studies have supported this effect of computer literacy [38,40,41,53,60] and internet literacy [61,62].

The last challenge was the lack of using internet or computers. This challenge may also be related to perceived ease of use, as those who rarely use computers and the internet may perceive the system difficult to use. Several previous studies showed similar findings regarding the effect of lack of computer use [43,46,62] and internet use on the adoption of ePHRs [39,43,54,63-65].

Regarding the fourth theme, participants determined the following characteristics of nonusers of Patient Online, which were consistent with findings of previous studies: older people [61,66-69], who rarely use GP services [55,66,68,70,71], who have low income [46,52,72,73], who live near the practice [70], and who have lower literacy levels [46,52,72,73].

In the last theme, participants justified their nonuse of the system by indicating their preference for personal contact with their GP. This was attributed to the perceived advantages of personal contact over the system. This factor was found in other studies [40,51,52].

### Strengths

This study enabled us to explore new factors that were not examined by the quantitative part of the study (eg, lack of awareness) and previous studies (eg, lack of trust). Furthermore, this study allowed us, to some extent, to support and explain some relationships proposed in the quantitative study (eg, performance expectancy, perceived privacy, security).

To the best of our knowledge, this study had the largest sample size in comparison with all qualitative studies on this topic. This allowed us to explore a wide range of patients’ perspectives on the adoption of ePHRs.

### Limitations

This study collected data from 4 GPs implementing the same ePHR (ie, SystemOnline), which may limit the generalizability of this study to other practices implementing other ePHRs (ie, Patient Access, Patient Services, The Waiting Room, Engage Consult, and Evergreen Life/i-Patient). However, it should be noted that all these systems provide the same services to the patients (ie, booking appointments, requesting prescription refills, and viewing health records), and no participant had used any of them before. As a result, the participants in this study were unlikely to have made comparisons between the different systems.

Although the qualitative data collected by an open-ended question helped in exploring factors affecting patients’ use of Patient Online, such data may not be equivalent to qualitative data collected by interviews or focus groups. Thus, we could not deeply understand the adoption process of Patient Online. However, this qualitative analysis did not aim to understand in depth the phenomenon of interest; rather, it aimed only to help in identifying other factors not included in the model and explaining the findings of the quantitative study. As answering the open-ended question was voluntary, there may be an element of self-selection.

As the open-ended question was put after closed-ended questions, participants’ answers to the open-ended question may be influenced by this order. This order was based on researchers’ recommendations that questionnaires should start with the most interesting and easy-to-answer questions, and open-ended and demographic questions should be presented at the end of the questionnaire [74-76].

### Practical Implications

We believe that adoption of GP online services will significantly increase in the future, given that many factors identified in this study will be automatically and considerably mitigated by time. Specifically, the proportion of patients who are more comfortable with the use of computers, smartphones, electronic systems, and the internet will increase in the future given their increased spread over the world. Thus, these services may be desired and expected by patients. However, developers, marketers, and GPs still play a crucial role in increasing the adoption of GP online services.

During system development, patients should be involved in the process to identify the features that make the system useful and easy to use. Some participants pointed out that the system will be useful when it allows them to book walk-in appointments, communicate with their doctors, and select the required doctor. As Patient Online currently enables patients to choose the required doctor, developers should consider adding these services, which are provided by many ePHRs (eg, MyChart, MyHealtheVet, Patient Gateway) [46,66,77]. Furthermore, users of such systems should be informed and reassured about the different security measures that are in place (eg, strong firewalls, encouragement to use complex and long passwords), and it should be made clear that the provision of GP online services is strictly controlled by legislation to safeguard personal data. To ease logging on to the system, developers should develop a system that allows patients to access it through their fingerprints or face recognitions, instead of using complex usernames and passwords. It is noteworthy that the NHS App, which has been recently developed, is the only system that enables patients to access GP online services using fingerprints or face recognitions [32].

To increase the awareness of the system, its functionality, and its benefits, marketers should improve their publicity through different channels, such as public media (eg, television, radio, newspapers, magazines), social media (eg, Facebook, Twitter, YouTube), emails, mails, automated messages on the practices’ telephone system, and advertisements in general public areas (eg, shopping centers, health care settings, highway streets, universities). Face-to-face communication is considered as one of the most effective channels in marketing to persuade potential adopters to adopt an innovation [78,79]. Thus, all staff in practice (eg, physicians, nurses, receptionists) should offer the system to patients during their visits. GP staff may not be keen on publicizing online services because of a lack of incentives and time. Therefore, consideration should be given to providing incentives and resources for GPs to increase patients’ awareness of GP online services.

Although patients have been recently enabled to sign up in the GP online services without visiting their surgeries through only the NHS App [80], they still need to visit their surgeries in person to register to use GP online services provided by other systems (eg, SystemOnline, Patient Access). To ease signing up in these systems, GPs should allow patients to register on web or through phone and make the signing up procedure a part of patient registration in the practice. GPs may enhance patients’ perceptions of usefulness, ease of use of the system, and their trust in it by helping them in using a beta version of the system through a computer in a waiting room. GPs should provide online assistance, technical support, manuals, and training to allow patients to solve any technical issues that face them when using the system, thereby decreasing their technical concerns. GPs should collaborate with other parties (eg, Patient Online providers and government bodies) to provide computers and/or internet access at affordable prices for those who do not have them and cannot afford them. Given that many UK GPs report being overstretched and limited funding has been provided to support the rollout of GP online services, consideration should be given to providing incentive programs (eg, Meaningful Use policy as issued by the US government). Incentive programs could be used to encourage GPs to publicize their online services and encourage patients to use them.

### Recommendations for Future Research

As this study could not provide a deep understanding of the adoption process of Patient Online, a deeper understanding of the adoption of online services could be gained through further qualitative work using interviews or focus groups. Several factors were revealed in this analysis but were not part of the conceptual model in the quantitative study, namely, awareness of Patient Online, lack of trust in the system, difficulty registering, disability, lack of use of GP services, and distance to the GPs. Future studies should consider adding these factors to the model and quantitatively examine them. Finally, more research is needed to identify the factors affecting the continuing use, as long-term viability and eventual success of information technology count on its continuing use more than initial use [81-83].

### Conclusions

This research explored patients’ perspectives regarding factors influencing their use of Patient Online. We found about 20 factors grouped into 6 themes. The findings of this study supported the findings of the quantitative study (eg, performance expectancy, effort expectancy, perceived privacy). This study found new factors that were not examined by the quantitative part of the study (eg, lack of awareness) and previous studies (eg, lack of trust).

The challenges and concerns that impede the use of Patient Online seem to be greater than the facilitators that encourage its use. To foster use, several practical implications were suggested: Patient Online should be useful, easy to use, secure, and easy to access; different channels should be used to increase the awareness of the system; and GPs should ease registration with the system and provide manuals, training sessions, and technical support. More research is needed to quantitatively assess the effect of the new factors found in this study (eg, lack of trust, difficulty registering with Patient Online) and factors affecting continuing use of the system.

## Appendix

Multimedia Appendix 1 Questionnaire.

## Abbreviations

ePHR: electronic personal health record
GP: general practice
NHS: National Health Service
SCR: summary care record
